# Supplementary material for: Examining human–carnivore interactions using a socio-ecological framework: sympatric wild canids in India as a case study
Source: R Soc Open Sci. 2019 May 29;6(5):182008. doi: 10.1098/rsos.182008 (PMC6549949; doi:10.1098/rsos.182008)
Supplement: Model comparisons [file rsos182008supp1.docx]

**Table S1** Model comparisons for probability of presence (ψ) and detectability (*p*) for five carnivores in the Kanha–Pench forest landscape, 2015–2016. For each species, the first set of models are for estimating detectability while the covariate structure for occupancy parameter is held constant; the second set of models are for estimating occupancy probability, retaining the top-ranked covariate(s) for detectability. The top five models (based on AIC ranks) are presented for all candidate sets. Covariate codes are explained in the footnote.

| **Model** | **AIC** | **ΔAIC** | **AIC**  **weight** | **Model Likelihood** | **Parameters** | **Deviance** |
| --- | --- | --- | --- | --- | --- | --- |
| **wolf (*p*)*** |  |  |  |  |  |  |
| ψ *(lstk+ndvi+scrb+rugg), p(rugg)* | 253.41 | 0 | 0.2385 | 1 | 7 | 239.41 |
| ψ *(lstk+ndvi+scrb+rugg), p(rugg+scrb+reps)* | 253.81 | 0.4 | 0.1952 | 0.8187 | 9 | 235.81 |
| ψ *(lstk+ndvi+scrb+rugg), p(rugg+reps)* | 254.66 | 1.25 | 0.1276 | 0.5353 | 8 | 238.66 |
| ψ *(lstk+ndvi+scrb+rugg), p(rugg+scrb+reps+dogs)* | 255.03 | 1.62 | 0.1061 | 0.4449 | 10 | 235.03 |
| ψ *(lstk+ndvi+scrb+rugg), p(rugg+lstk)* | 255.19 | 1.78 | 0.0979 | 0.4107 | 8 | 239.19 |
|  |  |  |  |  |  |  |
| **wolf (ψ)** |  |  |  |  |  |  |
| ψ *(ndvi+scrb+rugg), p(rugg)* | 251.47 | 0 | 0.264 | 1 | 6 | 239.47 |
| ψ *(ndvi+scrb), p(rugg)* | 253.33 | 1.86 | 0.1042 | 0.3946 | 5 | 243.33 |
| ψ *(lstk+ndvi+scrb+rugg), p(rugg)* | 253.41 | 1.94 | 0.1001 | 0.3791 | 7 | 239.41 |
| ψ *(dogs+ndvi+scrb+rugg), p(rugg)* | 253.46 | 1.99 | 0.0976 | 0.3697 | 7 | 239.46 |
| ψ *(ndvi), p(rugg)* | 253.47 | 2 | 0.0971 | 0.3679 | 4 | 245.47 |
|  |  |  |  |  |  |  |
| **dhole (*p*)*** |  |  |  |  |  |  |
| ψ *(.), p(fcov)* | 99.29 | 0 | 0.3722 | 1 | 3 | 93.29 |
| ψ *(.), p(fcov+smbr)* | 100.05 | 0.76 | 0.2545 | 0.6839 | 4 | 92.05 |
| ψ *(.), p(fcov+chtl)* | 101.29 | 2 | 0.1369 | 0.3679 | 4 | 93.29 |
| ψ *(.), p(fcov+chtl+smbr)* | 101.58 | 2.29 | 0.1184 | 0.3182 | 5 | 91.58 |
| ψ *(.), p(reps)* | 102.54 | 3.25 | 0.0733 | 0.1969 | 3 | 96.54 |
|  |  |  |  |  |  |  |
| **dhole (ψ)** |  |  |  |  |  |  |
| ψ *(lstk), p(fcov)* | 98.24 | 0 | 0.1677 | 1 | 4 | 90.24 |
| ψ *(lstk+chtl), p(fcov)* | 98.37 | 0.13 | 0.1572 | 0.9371 | 5 | 88.37 |
| ψ *(lstk+fcov), p(fcov)* | 98.39 | 0.15 | 0.1556 | 0.9277 | 5 | 88.39 |
| ψ *(lstk+chtl+fcov), p(fcov)* | 98.56 | 0.32 | 0.1429 | 0.8521 | 6 | 86.56 |
| ψ *(fcov), p(fcov)* | 98.67 | 0.43 | 0.1353 | 0.8065 | 4 | 90.67 |
|  |  |  |  |  |  |  |
| **jackal (*p*)** |  |  |  |  |  |  |
| ψ *(fcov+scrb+agri), p(scrb+agri)* | 1052.48 | 0 | 0.4784 | 1 | 9 | 1034.48 |
| ψ *(fcov+scrb+agri), p(dogs+agri+scrb)* | 1053.38 | 0.9 | 0.305 | 0.6376 | 10 | 1033.38 |
| ψ *(fcov+scrb+agri), p(dogs)* | 1055.24 | 2.76 | 0.1204 | 0.2516 | 8 | 1039.24 |
| ψ *(fcov+scrb+agri), p(.)* | 1056.51 | 4.03 | 0.0638 | 0.1333 | 7 | 1042.51 |
| ψ *(fcov+scrb+agri), p(agri)* | 1058.87 | 6.39 | 0.0196 | 0.041 | 8 | 1042.87 |
|  |  |  |  |  |  |  |
| **jackal (ψ)** |  |  |  |  |  |  |
| ψ *(scrb+rugg), p(scrb+agri)* | 1049.89 | 0 | 0.2393 | 1 | 8 | 1033.89 |
| ψ *(scrb), p(scrb+agri)* | 1050.74 | 0.85 | 0.1564 | 0.6538 | 7 | 1036.74 |
| ψ *(scrb+rugg+fcov), p(scrb+agri)* | 1051.04 | 1.15 | 0.1346 | 0.5627 | 9 | 1033.04 |
| ψ *(scrb+rugg+agri), p(scrb+agri)* | 1051.83 | 1.94 | 0.0907 | 0.3791 | 9 | 1033.83 |
| ψ *(scrb+fcov), p(scrb+agri)* | 1052.59 | 2.7 | 0.062 | 0.2592 | 8 | 1036.59 |
|  |  |  |  |  |  |  |
| **fox (*p*)** |  |  |  |  |  |  |
| ψ *(agri+ndvi+rugg+scrb), p(rugg)* | 414.08 | 0 | 0.212 | 1 | 9 | 396.08 |
| ψ *(agri+ndvi+rugg+scrb), p(scrb)* | 414.15 | 0.07 | 0.2047 | 0.9656 | 9 | 396.15 |
| ψ *(agri+ndvi+rugg+scrb), p(agri)* | 414.7 | 0.62 | 0.1555 | 0.7334 | 9 | 396.7 |
| ψ *(agri+ndvi+rugg+scrb), p(dogs)* | 415.34 | 1.26 | 0.1129 | 0.5326 | 9 | 397.34 |
| ψ *(agri+ndvi+rugg+scrb), p(ndvi)* | 415.44 | 1.36 | 0.1074 | 0.5066 | 9 | 397.44 |
|  |  |  |  |  |  |  |
| **fox (ψ)** |  |  |  |  |  |  |
| ψ *(ndvi), p(rugg)* | 408.4 | 0 | 0.1754 | 1 | 6 | 396.4 |
| ψ *(agri), p(rugg)* | 408.95 | 0.55 | 0.1333 | 0.7596 | 6 | 396.95 |
| ψ *(dogs), p(rugg)* | 409.55 | 1.15 | 0.0987 | 0.5627 | 6 | 397.55 |
| ψ *(scrb), p(rugg)* | 409.56 | 1.16 | 0.0982 | 0.5599 | 6 | 397.56 |
| ψ *(rugg), p(rugg)* | 409.59 | 1.19 | 0.0968 | 0.5516 | 6 | 397.59 |
|  |  |  |  |  |  |  |
| **hyena (*p*)*** |  |  |  |  |  |  |
| ψ *(scrb+lstk+ndvi), p(reps)* | 150.66 | 0 | 0.1851 | 1 | 6 | 138.66 |
| ψ *(scrb+lstk+ndvi), p(scrb)* | 150.67 | 0.01 | 0.1841 | 0.995 | 6 | 138.67 |
| ψ *(scrb+lstk+ndvi), p(dogs)* | 150.72 | 0.06 | 0.1796 | 0.9704 | 6 | 138.72 |
| ψ *(scrb+lstk+ndvi), p(rugg)* | 150.73 | 0.07 | 0.1787 | 0.9656 | 6 | 138.73 |
| ψ *(scrb+lstk+ndvi), p(scrb+dogs)* | 152.64 | 1.98 | 0.0688 | 0.3716 | 7 | 138.64 |
|  |  |  |  |  |  |  |
| **hyena (ψ)** |  |  |  |  |  |  |
| ψ *(lstk), p(reps)* | 147.44 | 0 | 0.3234 | 1 | 4 | 139.44 |
| ψ *(scrb+lstk+rugg), p(reps)* | 148.31 | 0.87 | 0.2093 | 0.6473 | 6 | 136.31 |
| ψ *(rugg+lstk), p(reps)* | 148.49 | 1.05 | 0.1913 | 0.5916 | 5 | 138.49 |
| ψ *(scrb+lstk), p(reps)* | 148.7 | 1.26 | 0.1722 | 0.5326 | 5 | 138.7 |
| ψ *(scrb+lstk+ndvi), p(reps)* | 150.66 | 3.22 | 0.0646 | 0.1999 | 6 | 138.66 |

chtl-chital abundance ; smbr- sambar abundance; fcov- forest cover; scrb- scrubland cover; agri- agricultural land; ndvi- Normalized Difference Vegetation Index; rugg- terrain ruggedness; dogs- abundance of free-ranging dogs; lstk- abundance of livestock; reps- number of surveyed replicates per site; * species for which data were fitted with standard single species models instead of the correlated-detections model; models do not include combinations of highly correlated covariates (*r* > |0.7|)

**Table S2** Model comparisons for probability of presence-only without depredation (ψ_p_), probability of depredation (ψ_d_), and associated detection probabilities (*p*_pp_*, p*_dd_*, p*_pd_) for three carnivores in the Kanha–Pench forest landscape, 2015–2016. The top five models (based on AIC ranks) are presented for each species. Covariate codes are explained in the footnote.

| **Model** | **AIC** | **ΔAIC** | **AIC**  **weight** | **Model Likelihood** | **Parameters** | **Deviance** |
| --- | --- | --- | --- | --- | --- | --- |
| **wolf** |  |  |  |  |  |  |
| ψ_p_ *(.),* ψ_d_ *(sett+goat), p*_pp_*(ints), p*_dd_*(ints), p*_pd_*(.)* | 439.07 | 0 | 0.2807 | 1 | 9 | 421.07 |
| ψ_p_ *(.),* ψ_d_ *(occp+sett+goat), p*_dd_*(ints), p*_dd_ *(ints), p*_pd_*(.)* | 439.54 | 0.47 | 0.2219 | 0.7906 | 10 | 419.54 |
| ψ_p_ *(.),* ψ_d_ *(scrb+sett+goat), p*_dd_*(ints), p*_dd_ *(ints), p*_pd_*(.)* | 439.84 | 0.77 | 0.191 | 0.6805 | 10 | 419.84 |
| ψ_p_ *(.),* ψ_d_ *(sett), p*_pp_*(ints), p*_dd_*(ints), p*_pd_*(.)* | 441.62 | 2.55 | 0.0784 | 0.2794 | 8 | 425.62 |
| ψ_p_ *(.),* ψ_d_ *(goat), p*_pp_*(ints), p*_dd_*(ints), p*_pd_*(.)* | 442.17 | 3.1 | 0.0596 | 0.2122 | 8 | 426.17 |
|  |  |  |  |  |  |  |
| **dhole** |  |  |  |  |  |  |
| ψ_p_ *(.),* ψ_d_ *(.), p*_pp_*(ints), p*_dd_*(ints), p*_pd_*(.)* | *327.87* | *0* | *0.1577* | *1* | *4* | *319.87* |
| ψ_p_ *(.),* ψ_d_ *(fcov), p*_pp_*(ints), p*_dd_*(ints), p*_pd_*(.)* | *327.93* | *0.06* | *0.153* | *0.9704* | *5* | *317.93* |
| ψ_p_ *(.),* ψ_d_ *(lstk), p*_pp_*(ints), p*_dd_*(ints), p*_pd_*(.)* | *328.08* | *0.21* | *0.1419* | *0.9003* | *5* | *318.08* |
| ψ_p_ *(.),* ψ_d_ *(occp), p*_pp_*(ints), p*_dd_*(ints), p*_pd_*(.)* | *328.27* | *0.4* | *0.1291* | *0.8187* | *5* | *318.27* |
| ψ_p_ *(.),* ψ_d_ *(fcov+lstk), p*_pp_*(ints), p*_dd_*(ints), p*_pd_*(.)* | *329.01* | *1.14* | *0.0892* | *0.5655* | *6* | *317.01* |
|  |  |  |  |  |  |  |
| **fox** |  |  |  |  |  |  |
| ψ_p_ *(.),* ψ_d_ *(ptry+sett), p*_pp_*(ints), p*_dd_*(ints), p*_pd_*(.)* | 642.15 | 0 | 0.1836 | 1 | 9 | 624.15 |
| ψ_p_ *(.),* ψ_d_ *(ptry+sett+occp), p*_pp_*(ints), p*_dd_*(ints), p*_pd_*(.)* | 642.77 | 0.62 | 0.1347 | 0.7334 | 10 | 622.77 |
| ψ_p_ *(.),* ψ_d_ *(occp), p*_pp_*(ints), p*_dd_*(ints), p*_pd_*(.)* | 643.19 | 1.04 | 0.1091 | 0.5945 | 8 | 627.19 |
| ψ_p_ *(.),* ψ_d_ *(ptry+occp), p*_pp_*(ints), p*_dd_*(ints), p*_pd_*(.)* | 643.35 | 1.2 | 0.1008 | 0.5488 | 9 | 625.35 |
| ψ_p_ *(.),* ψ_d_ *(ptry+sett+scrb), p*_pp_*(ints), p*_dd_*(ints), p*_pd_*(.)* | 643.35 | 1.2 | 0.1008 | 0.5488 | 10 | 623.35 |

fcov- forest cover; scrb- scrubland cover; rugg- terrain ruggedness; sett- area of human settlements; lstk- livestock abundance; goat- average goat-holding; ptry- average poultry-holding; occp- occupancy probability; ints- number of interviews per site; models do not include combinations of highly correlated covariates (*r* > |0.7|)

**Table S3** Model comparisons for probability of presence (ψ) and detectability (*p*) of free-ranging dogs in the Kanha–Pench forest landscape, 2015–2016. The first set of models are for estimating detectability while the occupancy parameter is held constant; the second set of models are for estimating occupancy probability, retaining the top-ranked covariate(s) for detectability. The top five models (based on AIC ranks) are presented. Covariate codes are explained in the footnote.

| **Model** | **AIC** | **ΔAIC** | **AIC**  **weight** | **Model Likelihood** | **Parameters** | **Deviance** |
| --- | --- | --- | --- | --- | --- | --- |
| **dog (*p*)** |  |  |  |  |  |  |
| ψ *(.), p(lstk)* | 908.26 | 0 | 0.4178 | 1 | 5 | 898.26 |
| ψ *(.), p(lstk +sett)* | 909.33 | 1.07 | 0.2447 | 0.5857 | 6 | 897.33 |
| ψ *(.), p(lstk +hpop)* | 910.21 | 1.95 | 0.1576 | 0.3772 | 6 | 898.21 |
| ψ *(.), p(sett)* | 910.97 | 2.71 | 0.1078 | 0.2579 | 5 | 900.97 |
| ψ *(.), p(.)* | 913.06 | 4.8 | 0.0379 | 0.0907 | 4 | 905.06 |
|  |  |  |  |  |  |  |
| **dog (ψ)** |  |  |  |  |  |  |
| ψ *(.), p(lstk)* | 908.26 | 0 | 0.2657 | 1 | 5 | 898.26 |
| ψ *(sett), p(lstk)* | 908.29 | 0.03 | 0.2618 | 0.9851 | 6 | 896.29 |
| ψ *(hpop), p(lstk)* | 909.15 | 0.89 | 0.1703 | 0.6408 | 6 | 897.15 |
| ψ *(lstk), p(lstk)* | 909.89 | 1.63 | 0.1176 | 0.4426 | 6 | 897.89 |
| ψ *(sett+lstk), p(lstk)* | 910.29 | 2.03 | 0.0963 | 0.3624 | 7 | 896.29 |

sett- area of human settlements; lstk- abundance of livestock; hpop- human population in each site; models do not include combinations of highly correlated covariates (*r* > |0.7|). sett and lstk obtained as described in the manuscript. hpop data for each site extracted from WorldPop (WorldPop. 2017. India 100m Population, Version 2. University of Southampton. DOI: 10.5258/SOTON/WP00532.)
